# O.2.3-8 Work engagement, need for recovery, and musculoskeletal symptoms – gender differences among office workers during the COVID-19 pandemic

**DOI:** 10.1093/eurpub/ckad133.134

**Published:** 2023-09-11

**Authors:** Luiz A Brusaca, Leticia Bergamin Januario, Dechristian França Barbieri, Marina Machado Cid, Ana Beatriz Oliveira

**Affiliations:** Department of Physical Therapy, Federal University of São Carlos, São Carlos, Brazil; Centre for Musculoskeletal Research, Department of Occupational Health Science and Psychology, University of Gävle, Gävle, Sweden; Department of Physical Therapy, Federal University of São Carlos, São Carlos, Brazil; Department of Industrial Engineering, Clemson University, Clemson, USA; augustobrusaca@gmail.com; Department of Physical Therapy, Federal University of São Carlos, São Carlos, Brazil; Department of Physical Therapy, Federal University of São Carlos, São Carlos, Brazil

## Abstract

**Purpose:**

The COVID-19 pandemic has changed work conditions and personal lives of countless workers due to physical distancing, and recommendations to work from home (WFH), especially among office workers. This study aimed to investigate whether male and female office workers differed in terms of work engagement, need for recovery, and intensity of musculoskeletal symptoms, and if gender differences were still present depending on the workplace, i.e., WFH or working at the office (WAO).

**Methods:**

This cross-sectional study collected data from 56 male (age 36.1 ± 10.3 years) and 60 female (age 35.2 ± 8.9 years) office workers between September 2020–June 2021. Participants were asked to answer an online self-administrated survey containing the Utrecht Work Engagement Scale (total score [0-6]; subscales of vigor, dedication, and absorption not shown), the Need For Recovery Scale (0-100), and a modified version of the Nordic Musculoskeletal Questionnaire, considering the pain intensity for each region with a numerical rating scale (0-10). Differences between gender (male *vs.* female) were examined using linear regressions adjusted for age and body mass index, considering the total population, those exclusively WFH (34♀ and 39♂) and those exclusively WAO (22♀ and 21♂).

**Results:**

Female office workers reported worse conditions during the pandemic compared with male workers: they had less engagement with their work (β: –0.65 [95% CI: –1.09; –0.21]), greater need for recovery (16.00 [8.69; 23.31]), and higher pain intensity in the neck (1.44 [0.35; 2.54]), shoulders (2.13 [1.13; 3.13]), upper back (1.41 [0.46; 2.35]), wrists/hands (1.65 [0.65; 2.65]), and hips/thighs (0.71 [0.05; 1.38]). Sensitivity analyses showed similar results when workers were exclusively WAO, except that there was no difference in musculoskeletal symptoms. When WFH, females had a greater need for recovery and higher pain intensity in all regions found in the main analysis when compared with male workers, but no difference was found in work engagement.

**Conclusions:**

Female office workers had less work engagement, more need for recovery, and more self-reported pain intensity than male workers. Whether this relates to additional unpaid work or to other factors is not clear, and we encourage further research to resolve this important issue.

